# Supplementary material for: Congenital Myopathy as a Phenotypic Expression of CACNA1S Gene Mutation: Case Report and Systematic Review of the Literature
Source: Genes (Basel). 2023 Jun 28;14(7):1363. doi: 10.3390/genes14071363 (PMC10379235; doi:10.3390/genes14071363)
Supplement: Supplementary file 1 [file genes-14-01363-s001.zip › genes-2441847-supplementary.pdf]

	Case	Mutations	Position in CACNA1S gene	Position in Cav 1.1	Phenotype	Varsome	CADD Score	Clin Var
**Our case**	1	c.1394-2A>G	intron 10 of 43 position 5056 of 5057	T: Helical, S2 of II domain	Mild	Likely Pathogenetic	34	-
		c.1724T>C, p.Leu575Pro	exon 12 of 44 position 105 of 208	T: Helical, S5 of II domain		Uncertain Significance	31	Uncertain Significance
**Schartner et al.**	2	c.1189_1190del, p.Ser397Pro	exon 9 of 44 position 39-40 of 82	C: between I and II domains	Mild	Pathogenetic	25.1	Pathogenetic
		c.4967del, p.Leu1656Arg	exon 40 of 44 position 170 of 251	C: afte IV domain		Pathogenetic	23.5	Pathogenetic
	3	c.4453C>T, p.Gln1485*	exon 37 of 44 position 12 of 102	C: afte IV domain	Mild	Uncertain Significance	20.5	Pathogenetic
		c.4967del, p.Leu1656Arg	exon 40 of 44 position 170 of 251	C: afte IV domain		Pathogenetic	23.5	Pathogenetic
	4	c.825C>A, p.Phe275Leu	exon 6 of 44 position 131 of 206	E: between S5 and S6 of I domain	Mild	Uncertain Significance	20.5	Pathogenetic
		c.2371delC, p.Leu791Cys	exon 18 of 44 position 11 of 130	C: between II and III domains		Pathogenetic	-	Pathogenetic
	5	c.825C>A, p.Phe275Leu	exon 6 of 44 position 131 of 206	E: between S5 and S6 of I domain	Moderate	Uncertain Significance	20.5	Pathogenetic
		c.2371delC, p.Leu791Cys	exon 18 of 44 position 11 of 130	C: between II and III domains		Pathogenetic	19.27	Pathogenetic
	6	c.298G>T, p.Glu100Lys	exon 3 of 44 position 40 of 140	T: Helical, S2 of I domain	Severe	Likely Pathogenetic	39	-
		c.3795G>T, p.Gln1265His	exon 30 of 44 position 129 of 129	C: between S4 and S5 of IV domain		Pathogenetic	36	Likely Pathogenetic
	7	c.2225C>A, p.Pro742Gln	exon 16 of 44 position 68 of 70	C: between II and III domains	Mild	Uncertain Significance	33	Pathogenetic
	8	c.2225C>A, p.Pro742Gln	exon 16 of 44 position 68 of 70	C: between II and III domains	Mild	Uncertain Significance	33	Pathogenetic
	9	c.2225C>A, p.Pro742Gln	exon 16 of 44 position 68 of 70	C: between II and III domains	Mild	Uncertain Significance	33	Pathogenetic
	10	c.2224C>T, p.Pro742Ser	exon 16 of 44 position 67 of 70	C: between II and III domains	Severe	Uncertain Significance	24.4	Pathogenetic
	11	c.4099C>G, p.Leu1367Val	exon 33 of 44 position 146 of 160	T: Helical, S6 of IV domain	Mild	Uncertain Significance	27.8	Uncertain Significance
	12	c.4099C>G, p.Leu1367Val	exon 33 of 44 position 146 of 160	T: Helical, S6 of IV domain	Mild	Uncertain Significance	27.8	Uncertain Significance
**Yis et al.**	13	c.2366G>A, p.Arg789His *homo	exon 18 of 44 position 6 of 130	C: between II and III domains	Severe	Uncertain Significance	28	Uncertain Significance
	14	c.2366G>A, p.Arg789His *homo	exon 18 of 44 position 6 of 130	C: between II and III domains	Severe	Uncertain Significance	28	Uncertain Significance
	15	c.2366G>A, p.Arg789His *homo	exon 18 of 44 position 6 of 130	C: between II and III domains	Severe	Uncertain Significance	28	Uncertain Significance
**Morales et al.**	16	c.2970G>A, p.Trp990*	exon 24 of 44 position 64 of 147	E: between S5 and S6 of III domain	Moderate	Pathogenetic	48	Pathogenetic
		c.5104C>T, p.Arg1702*	exon 41 of 44 position 56 of 86	C: afte IV domain		Pathogenetic	36	Conflicting
	17	c.2447T>G, p.Leu816Arg	exon 18 of 44 position 87 of 130	T: Helical, S1 of III domain	Moderate	Uncertain Significance	26.5	-
**Hunter et al.**	18	c.4947delA, p.Gln1649Gln	exon 40 of 44 position 150 of 251	C: afte IV domain	Moderate	Pathogenetic	-	Pathogenetic
		c.3795G>T, p.Gln1265His	exon 30 of 44 position 129 of 129	C: between S4 and S5 of IV domain		Pathogenetic	36	Likely Pathogenetic
**Francois-Heude et al.**	19	c.2618del, p.Leu873Arg	exon 20 of 44 position 68 of 107	T: Helical, S3 of III domain	Moderate	Likely Pathogenetic	28.9	-
		c.5104C>T, p.Arg1702*	exon 41 of 44 position 56 of 86	C: afte IV domain		Pathogenetic	36	Conflicting
**Mauri et al.**	20	c.3364 T > C, p.Tyr1122His	exon 26 of 44 position 109 of 159	T: Helical, S1 of IV domain	Mild	Uncertain Significance	29.7	-
**Ravenscroft et al.**	21	c.665T>A, p.Met222Lys	exon 5 of 44 position 124 of 153	E: between S5 and S6 of I domain	Severe	Uncertain Significance	27.9	Uncertain Significance
		c.2365C>T, p.Arg789Cys	exon 18 of 44 position 5 of 130	C: between II and III domains		Uncertain Significance	31	Uncertain Significance
	22	c.665T>A, p.Met222Lys	exon 5 of 44 position 124 of 153	E: between S5 and S6 of I domain	Severe	Uncertain Significance	27.9	Uncertain Significance
		c.2365C>T, p.Arg789Cys	exon 18 of 44 position 5 of 130	C: between II and III domains		Uncertain Significance	31	Uncertain Significance
**Grunseich C. et al.**	23	c.2725A>G, p.Asn909Ser	exon 21 of 44 position 68 of 88	T: Helical, S4 of III domain	Mild	Uncertain Significance	28.5	-
**Kausthubham et al.**	24	c.4316G>T, p.Cys1439Phe	exon 35 of 44 position 75 of 97	C: afte IV domain	Severe	Uncertain Significance	27.4	Uncertain Significance

Table S1: Position in CACNA1S gene and in Cav 1.1, Phenotype (mild, moderate, severe), Varsome grade (https://varsome.com/) [26], CADD score (Combined Annotation Dependent

Depletion score, https://cadd.gs.washington.edu/snv) [29] and Clin Var index (https://www.ncbi.nlm.nih.gov/clinvar/) of variants in *CACNA1S* gene individuated in 24 patients.
